# Hydrated Electron Dynamics and Stimulated Raman Scattering in Water Induced by Ultrashort Laser Pulses

**DOI:** 10.3390/molecules29061245

**Published:** 2024-03-11

**Authors:** Jun Tang, Zhongyang Wang

**Affiliations:** 1 Shanghai Advanced Research Institute, Chinese Academy of Sciences, Shanghai 201210, China; tangjun@sari.ac.cn; 2 School of Microelectronics, University of Chinese Academy of Sciences, Beijing 100049, China

**Keywords:** laser-induced breakdown, filaments, excess electrons, hydrated electrons, stimulated Raman scattering, Raman spectrum

## Abstract

For this study, we employed intense 400 nm, 100 fs pulses linearly propagated through a 50 cm water medium, initially self-stretching the excitation pulses to 2.50 ps. Subsequently, the self-stretched 2.50 ps pulses were focused into deionized water, and we conducted transient absorption experiments to measure and investigate the dynamics of hydrated electrons in water. The excess electrons generated were injected into the hydrogen bond network of the water cluster, leading to the observation of saturated hydrated electrons. Additionally, we observed the emergence of the forward stimulated Raman scattering (SRS) of water molecules. We report the experimental observation of a weak forward SRS emission at 463 nm (corresponding to 3400 cm^−1^), indicative of the ordinary OH stretching vibration in the liquid phase. Moreover, we observed an intense forward SRS emission at 460 nm in water, corresponding to two anomalous Raman shifts at 3260 cm^−1^ and 3355 cm^−1^. These anomalous Raman shifts resulted from changes in the hydrogen bond network structure. We determine that the formation of not fully hydrated and saturated hydrated electrons plays a crucial role in producing this phenomenon.

## 1. Introduction

The study of excess electrons in liquids has attracted much interest from both chemists and physicists since the first observation of solvated electrons in liquid ammonia [1]. In 1952, Stein, G. and Hart, E.J. et al. first proposed the existence of excess electrons in aqueous solutions, referred to as hydrated electrons ($e_{aq}^{-}$) [2,3]. Ten years later, Hart, E.J., Boag, J.W., and Keene, J. P. et al. discovered the broad absorption spectrum of hydrated electrons in radiolysis studies using pulsed electron beams for the first time [4,5,6]. Since their discovery in 1962, hydrated electrons have been extensively studied for their ionization, solvation, and relaxation dynamics. In the past few decades, hydrated electrons have also been observed in the context of laser-induced breakdown (LIB) through the use of intense pulsed lasers in liquid water [7,8,9,10]. LIB is a complex nonlinear process involving the self-induced Kerr effect, multiphoton absorption, and electron formation. This process sometimes accompanies laser filamentation and stimulated Raman scattering (SRS) in water molecules. These generated excess electrons are injected into the hydrogen bond network of the water cluster [11], which is believed to be trapped in a cavity space bounded by six water molecules ${\left(H_{2}O\right)}_{6}^{-}$ [12], forming the hydrated electrons in water [12,13].

Previous studies have shown that hydrated electrons play an important role in radiation chemistry, physical chemistry, photochemistry, and electron transfer [14,15,16]. Although extensive research has been conducted on the threshold of the LIB process excited with picosecond and femtosecond pulses, as well as the relaxation dynamics of hydrated electrons in water [10,14,17,18,19,20,21,22], the interplay between excess electrons and the hydrogen bond network structure of water clusters is still unclear. Only recently, when Hiroharu Yui and Tsuguo Sawada utilized an intense 532 nm, 40 ps pulse focused into water, they observed not only a normal Raman shift in the forward SRS at 3400 cm^−1^, corresponding to the ordinary OH stretching vibration of water molecules in the liquid phase [23], but also an anomalous Raman shift in the backward SRS at 3270 cm^−1^ and 3360–3380 cm^−1^ [7,8,9], which they attributed to the formation of water clusters through the hydrated electrons changing the hydrogen bond network structure, resulting in anomalous Raman shift appearances in the backward region [7,8,9].

In this article, we report an anomalous Raman shift in forward SRS, arising from saturated hydrated electrons within filaments induced by broadband pulse excitation in water. Using 400 nm, 100 fs pulses linearly propagated into 0.5 m water medium, the excitation 400 nm pulses were first self-stretched to 2.50 ps, following which the self-stretched 400 nm pulse was weakly focused into 1 cm long deionized water. The induced excess electrons were generated and subsequently saturated within the 0.50 ps pulse front. These saturated excess electrons were then injected into the hydrogen bond network of the water cluster, forming saturated hydrated electrons in water. We report the experimental observation of forward SRS emission in liquid water. A weak forward Stokes emission at 463 nm was observed at low input pump energy, corresponding to the Raman shift of 3400 cm^−1^ and indicative of the ordinary OH stretching vibration of water molecules in the liquid. As the input pump energies increased, we also observed an intense forward SRS emission at 460 nm in water, corresponding to two anomalous Raman shifts at 3260 cm^−1^ and 3350 cm^−1^. These anomalous Raman shifts arose from the changes in the hydrogen bond network structure, and we determined that the formation of saturated hydrated electrons plays a key role in generating this phenomenon.

## 2. Result

### 2.1. The Dynamics of Hydrated Electrons in Water

**Figure 1:**
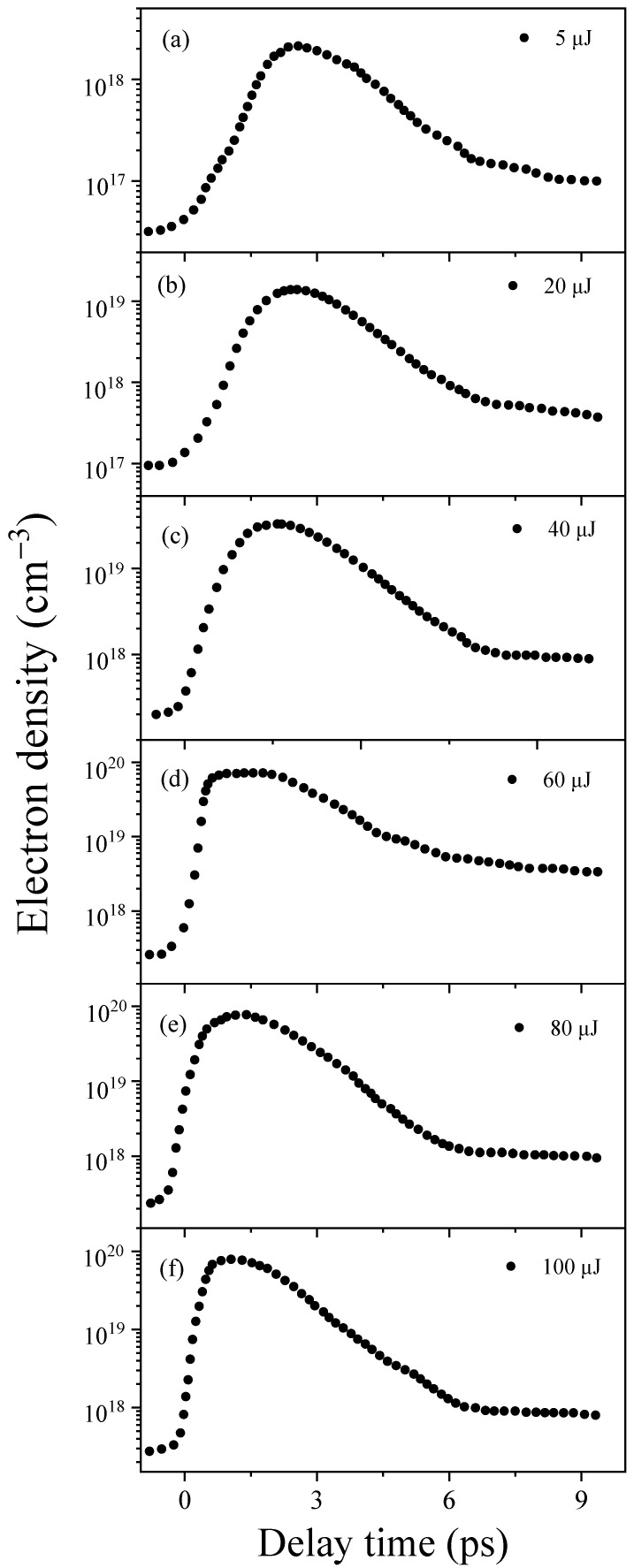
The transient dynamics of the hydrated electron density with various pumping energies in 1 cm of deionized water. Panels (**a**–**f**) depict the hydrated electron density corresponding to 5, 20, 40, 60, 80, and 100 µJ, respectively.

During the LIB process in water, excess electrons are generated, which subsequently form hydrated electrons. To investigate their dynamics, transient absorption experiments were conducted in deionized water (L = 1 cm). The experiments involved various pumping energies with a stretched 400 nm, 2.50 ps pump pulse (the measured stretched pulse width, as shown in Figure S2) and probing with a 720 nm, 100 fs pulse around the absorption peak of the hydrated electrons [24]. Specific experimental details of transient absorption experiments are illustrated in Figure 4. The measured electron density can be derived from the Lambert–Beer law ($\Delta T/T=\sigma_{a}L\rho$), where $\sigma_{a}=1.4\times 10^{-18}$ cm^2^ is the absorption cross-section of the hydrated electrons [24]. As shown in Figure 1, we measured the dynamics of hydrated electrons in water with different input 400 nm pump energies, ranging from 5 to 100 µJ. Despite variations in the input pump energy, we observed a rapid increase in electron density, followed by saturation and, ultimately, a gradual decay through two relaxation processes. This observation agrees with the picosecond plasma dynamics obtained using time-resolved imaging [10] and scattering techniques and using picosecond pulse radiolysis measurement in water [22].

The subsequent analysis primarily concentrates on the rising and saturation regions of dynamics of hydrated electrons, as shown in Figure 1. A detailed discussion of the relaxation process will be presented elsewhere. First, we observed an exponential increase in these electron densities. However, the time scale for the rising regions varied. When the input pump energies were 5 µJ, 20 µJ, 40 µJ, 60 µJ, 80 µJ, and 100 µJ, the corresponding rise times were 2.25 ps, 1.85 ps, 1.25 ps, 0.50 ps, 0.45 ps, and 0.43 ps, respectively. Subsequently, the generated excess electrons approached saturation regime within a time scale of approximately 2.5 to 3.0 ps, which is indicative of the electron density remaining stable throughout the entire pulse width (2.50 ps) without entering into relaxation. Furthermore, we observed variations in the maximum electron densities corresponding to different input pump energies in deionized water. Further details will be discussed later. Moreover, the generated saturated electrons were injected into the hydrogen bond network of the water cluster [11], which is believed to be trapped in a cavity space bounded by six water molecules ${\left(H_{2}O\right)}_{6}^{-}$ [12], forming the hydrated electrons in water [12,13]. However, the electrons generated during the rising regime were primarily utilized for collisional ionization to induce excess electrons rather than fully forming hydrated electrons.

**Figure 2:**
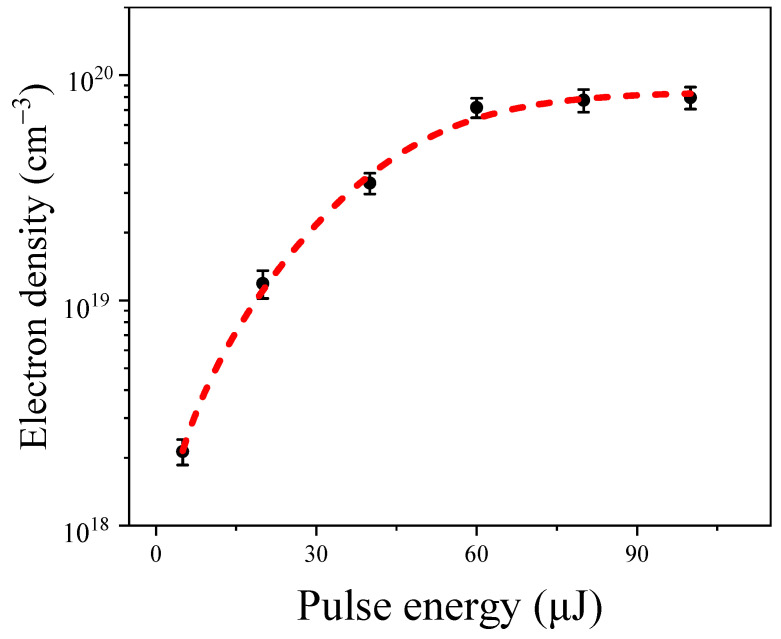
The hydrated electron density as a function of the input pulse energies in water, with error bars representing standard deviations. The red dotted line shows the fitting results according to Equation (2).

Figure 2 shows the variations in the maximum hydrated electron density under different input pump energies in water. Through analysis of the various intensity dependence data, we found that when the input pump pulse energy exceeded 5 µJ, the electron density increased rapidly with an increase in the input pump energy, approaching saturation at around 60 µJ. Specifically, for input pump energies of 5 µJ, 20 µJ, 40 µJ, 60 µJ, 80 µJ, and 100 µJ, the corresponding hydrated electron densities were 2.13 ± 0.27 × 10^18^ cm^−3^, 1.18 ± 0.17 × 10^19^ cm^−3^, 3.36 ± 0.35 × 10^19^ cm^−3^, 7.17 ± 0.71 × 10^19^ cm^−3^, 7.23 ± 0.89 × 10^19^ cm^−3^, and 7.59 ± 0.88 × 10^19^ cm^−3^, respectively. This phenomenon can be attributed to the balance of the formation of hydrated electrons [7] and their recombination [25]. The observed trend of electron density with increasing input pump energy was consistent with simulated results using picosecond or nanosecond laser pulses in water [25].

When the input pulse energy exceeds the critical power ($P_{cr}=3.77\lambda^{2}/8\pi n_{0}n_{2}$) [26], filamentation occurs, accompanied by the generation of electrons in water. The evolution of the free-electron density is described by the following equation [25,27,28]:

(1) $$\frac{\partial \rho }{\partial t}=\frac{\sigma }{n_{0}^{2}E_{g}}I\rho +\frac{\beta^{\left(K\right)}}{K\hbar \omega_{p}}I^{K}-\eta \rho^{2}$$

The first term on the right-hand side of Equation (1) describes the cascade ionization, where $n_{0}=1.34$ is the refractive index of water, $E_{g}=6.5$ eV is the ionization energy of water for a 400 nm pump pulse, $I_{p}=\left(E_{in}/\tau_{p}\right)/\pi r_{0}^{2}$ is the laser intensity (here, $E_{in}$ is the input pump energies, $\tau_{p}=2.50$ ps is the pulse width of the pump beam, and $r_{0}=40\pm 10$ μm is the radius of the filaments, respectively), and $\sigma =\left(e^{2}\tau_{c}n_{0}/m_{e}\varepsilon_{0}c\right)/\left(1+\omega_{0}^{2}\tau_{c}^{2}\right)$ is the cross-section for inverse bremsstrahlung (here, $\tau_{c}=10^{-14}$ s is the collision time, $\varepsilon_{0}=8.85\times 10^{-12}$ F/m is the vacuum permittivity, and $m_{e}=9.1\times 10^{-31}$ kg is the mass of the electron). The second term describes multi-photon ionization (MPI), where $K=\langle E_{g}/\hbar \omega +1\rangle =3$ is the number of photons necessary to liberate an electron and $\beta^{\left(K\right)}=1.2\times 10^{-23}$ cm^3^/W^2^ is the multi-photon absorption cross-section for *K* = 3 photons, and the last term describes the recombination processes, where $\eta =2\times 10^{-9}$ cm^−3^/s. According to previous investigations, for long pulses (e.g., in the nanosecond and picosecond regime), breakdown is primarily caused by cascade ionization, while MPI dominates breakdown in the femtosecond regime [25,27,28]. Extending our interpretation further, we estimated that the electron density generated through the MPI process was $\rho_{MPI}=\beta^{\left(K\right)}I^{K}\tau_{p}/K\hbar \omega_{p}=3.3\times 10^{17}$ cm^−3^, approximately two orders of magnitude lower than the measured electron density (~10^19^ cm^−3^) within the filaments at 60 µJ input energy. Therefore, for the 2.50 ps excitation pulse, the phenomena observed in Figure 2 can be modeled with Equation (1) when neglecting the MPI process. In the following sections, we obtained induced electrons after the pulse excitations:

(2) $$\rho =\frac{\left(\sigma I/n_{0}^{2}E_{g}\right)}{\left[C/\exp(\sigma I/n_{0}^{2}E_{g})\right]+\eta }$$

where *C* is the background electron density. Equation (2) fits the experimental data well (shown as the red dotted line in Figure 2), with values of $C=2.9\pm 0.2\times 10^{10}$ cm^−3^ and $\eta =3.7\pm 0.4\times 10^{-9}$ cm^−3^/s. These values agree well with recent results in water [25,27,28].

### 2.2. The Forward SRS Spectrum in Water

**Figure 3:**
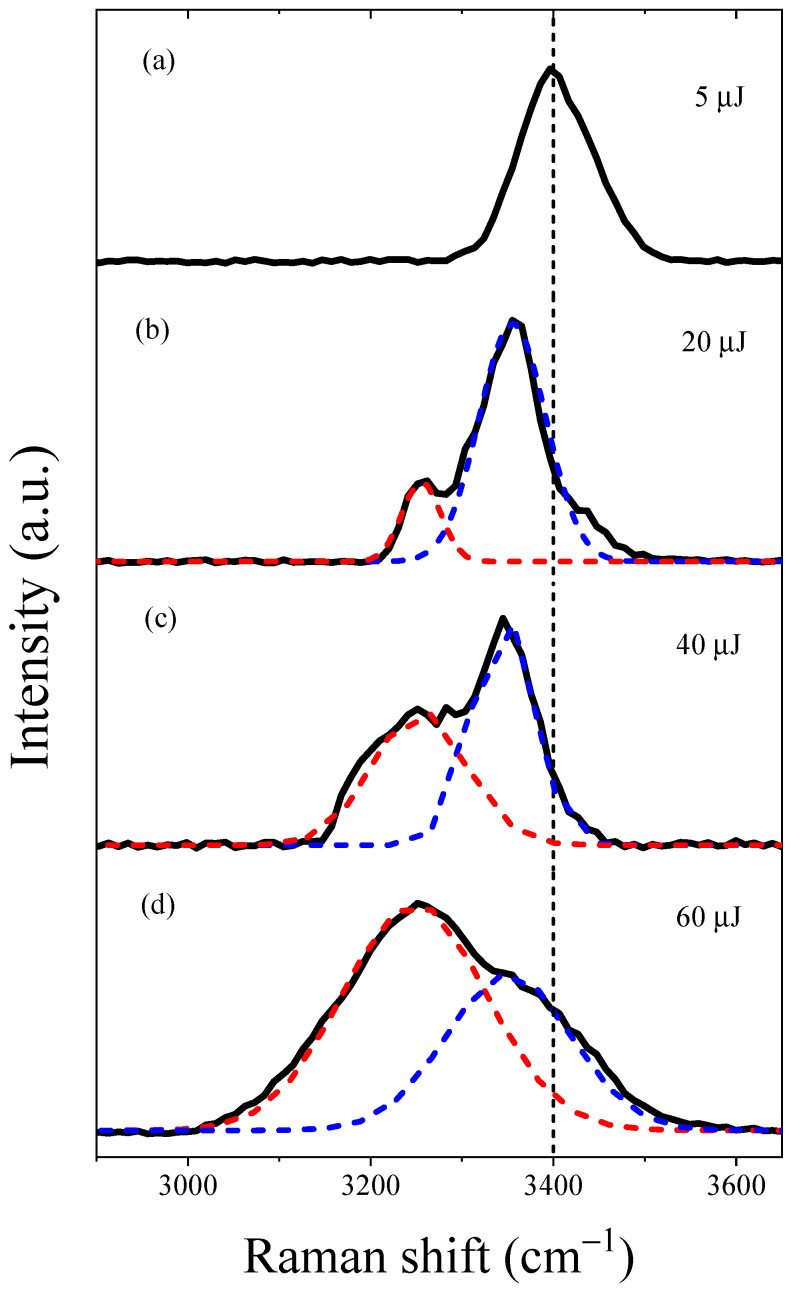
Forward SRS spectra were measured in deionized water with various input pump energies. (**a**) The Raman shifts of the forward Stokes emission was measured at 3400 cm^−1^ with an input energy of 5 µJ. (**b**–**d**) The forward Stokes emission showed two distinct peaks at 3260 cm^−1^ (red dotted line) and 3355 cm^−1^ (blue dotted line) for input pump energies 20 µJ, 40 µJ, and 60 µJ, respectively. The broken black dotted line in the figure represents the Raman shift (3400 cm^−1^) corresponding to the ordinary OH stretching vibrations of water molecules in the liquid phase.

During the LIB process accompanied with the generated excess electrons within the filaments in water, we also observed an interesting phenomenon of a bright-blue spot, indicative of SRS from the water molecules, in the forward direction along the optical axis. We measured the spectra of the output forward SRS conical emission under various input pump energies, as shown in Figure 3.

First, we observed the forward Stokes emission at 463 nm with an input pump energy of 5 µJ, exhibiting a full width at half-maximum (FWHM) linewidth of 4.3 nm (203 cm^−1^), as shown in Figure 3a. This linewidth was nearly consistent with the normal SRS linewidth of water (e.g., 200 cm^−1^) [29]. However, with increasing input pump energies, we observed an anomalous forward Stokes emission at 460 nm in water, as shown in Figure 3b–d. We noted a gradual increase in the FWHM linewidth of the forward Stokes emission at 460 nm. The FWHM linewidth was measured at 5.0 nm (236 cm^−1^), 6.2 nm (293 cm^−1^), and 7.5 nm (355 cm^−1^) for input pump energies of 15 µJ, 30 µJ, and 60 µJ, respectively. It is worth mentioning that the linewidths in Figure 3b–d are broader than the normal SRS linewidth in Figure 3a—a phenomenon generally considered to arise from the self-focusing effect of the Stokes pulse, indicating the high intensity of the Stokes pulse [30]. Furthermore, we also observed a weak second-order Stokes emission at 541 nm, revealing that the generation of strong Stokes acting as a pump to drive second-order SRS [31]. We provide a detailed explanation of the mechanism driving the generation of the second-order Stokes pulse elsewhere.

In the following analysis, we observed a significant change in the Raman shift of the forward Stokes emission in water as the input pump energies increased. Specifically, when the pulse energy of the pump beam was set at 5 µJ, we observed a weak forward Stokes emission at 463 nm with a Raman shift of 3400 cm^−1^, corresponding to the ordinary OH stretching vibration in the liquid phase [7,23], as shown in Figure 3a. However, with increasing input pump energy, we observed an intense anomalous forward Stokes emission at 460 nm that showed two characteristic peaks. These peaks were not observed under the low 5 µJ input pump energy during our experiment. We employed a double-peak fitting method to analyze the anomalous forward Stokes emissions. As shown in Figure 3b–d, we observed anomalous Raman shifts at 3355 cm^−1^ (blue dotted line) and 3260 cm^−1^ (red dotted line) in water. We observed that, with an increase in input energy, the intensity of the Raman shift at 3260 cm^−1^ gradually increased in comparison to that at 3355 cm^−1^. Specifically, when the input pump energy increased to 20 µJ, a weaker peak appeared at 3260 cm^−1^ and a stronger peak was observed at 3355 cm^−1^, as shown in Figure 3b. Subsequently, as the input pump energy increased to 40 µJ, the intensity of the anomalous Raman peak at 3260 cm^−1^ gradually increased in comparison to the stronger peak at 3355 cm^−1^, as shown in Figure 3c. Finally, upon reaching a saturation pump energy of 60 µJ, the intensity of the anomalous Raman peak at 3260 cm^−1^ exceeded that of the peak at 3355 cm^−1^, as shown in Figure 3d. Therefore, we can conclude that the anomalous Raman shift initially occurs at 3355 cm^−1^, followed by the appearance of the anomalous Raman shift at 3260 cm^−1^.

## 3. Discussion

In order to reveal the internal mechanism for the observed normal Raman shift at 3400 cm^−1^ and two characteristic anomalous forward Raman shifts at 3260 cm^−1^ and 3355 cm^−1^ (as shown in Figure 3), we propose that these Raman shifts result from the generation of excess electrons in water. Therefore, through analyzing the interplay between the generated excess electrons and Raman shifts, we determined the mechanism of the anomalous Raman shifts in liquid water. The specific analysis is as follows:

First, as shown in Figure 3a, the forward normal Raman shift at 3400 cm^−1^, obtained with a low 5 µJ input pump energy in water, corresponds to the ordinary OH stretching vibration in the liquid phase [7,23]. This observation suggests that the water molecules remained in a condensed state. We attribute this phenomenon to the generated low electron density of 2.13 × 10^18^ cm^−3^, which is approximately two orders of magnitude lower than the maximum electron density for a 60 µJ input pump energy. This generated low density cannot change the hydrogen bond network structure.

Second, the most interesting feature of the anomalous forward SRS spectrum in Figure 3 lies in its resemblance to the spectra of stable water anion clusters in the gas phase, despite the bandwidths of the two peaks being remarkably broader than those of the water anion clusters [32]. Within a water anion cluster, we can observe an additional generated electron strongly attaching to the positively polarized hydrogen atoms of water molecules and/or their dipoles through strong electrostatic interactions, consequently altering their molecular polarizability [8]. The similarity in spectral features suggests that the OH groups of water molecules—which are related to Raman enhancement—exist in a similar electrostatic environment within those water anion clusters. Therefore, we propose that the anomalous Raman shift results from the generated excess electrons being injected into the hydrogen bond network of the water cluster. This process leads to the formation of hydrated electrons, subsequently causing changes in the hydrogen bond network structure of the water cluster and including alterations in the cluster number.

More specifically, we observed anomalous Raman shifts in the forward SRS that exhibited two characteristic peaks at 3260 cm^−1^ and 3355 cm^−1^. These peaks were not observed at the low 5 µJ input pump energy during our experiment, as shown in Figure 3b–d. The specific analysis of these two anomalous Raman peaks is as follows:

(i) The anomalous Raman shift at 3350 cm^−1^ is attributed to the enhanced Raman cross-section of the OH groups in water molecules, corresponding to an increasing region of electron generation in the hydrated electron dynamics process. The distinctive features of the SRS spectrum and the similarities in the vibrational spectrum to the vibrational spectra of water anion clusters indicate that the excess electrons effectively generated in the forward region strongly enhanced the Raman cross-section of the water molecules. The enhancement of Raman scattering occurred in the presence of strong excitation laser fields, prior to the saturated region and recombination of excess electrons. Therefore, the excess electrons responsible for Raman enhancement were not fully hydrated electrons.
(ii) The anomalous Raman shift at 3260 cm^−1^ is attributed to the contribution of hydrated electrons in water. As shown in Figure 3b–d, with an increase in input pump energy, the intensity of the anomalous Raman shift at 3260 cm^−1^ gradually rises, corresponding to a non-linear increase in the hydrated electron density as incident energy rises, eventually reaching a saturation region. Furthermore, under the present experimental conditions, we noted that the saturated hydrated electrons reached about 7.17 × 10^19^ cm^−3^ under a 60 µJ input pump energy. The ratio of water molecules to electrons was about 465:1, indicating that the interaction with electrons drastically enhanced the Raman cross-section of the OH groups in the water molecules.

Hence, we can reasonably conclude that there are at least two distinctive hydration structures around the excess electrons in water. One corresponds to the SRS peak at 3260 cm^−1^, which is capable of strongly binding the hydrated electrons. The other corresponds to the SRS peak at 3355 cm^−1^, which is capable of weakly binding the excess electrons (not fully hydrated). Thus, we believe that the anomalous Raman shifts result from the generated excess electrons being injected into the hydrogen bond network of the water cluster, forming hydrated electrons and consequently altering the hydrogen bond network structure of the water cluster and the cluster number. The excess electrons generated—forming not fully hydrated or saturated hydrated electrons—play a key role in these phenomena.

## 4. Experiments

### 4.1. Experimental Measured the Dynamic of Hydrated Electrons in Water

**Figure 4:**
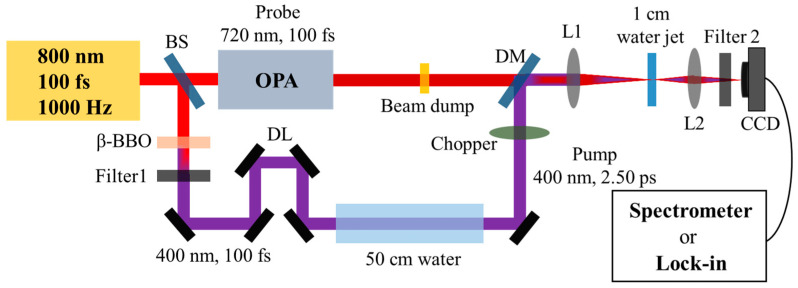
Schematic of the SRS experiment and the pump–probe experiment setup. Beam splitter (BS), beta barium borate (β-BBO) crystal, Filter 1 (short-pass filter, FESH0650), delay line (DL), 50 cm-long water cuvette, optical parametric amplifier (OPA) system, dichroic mirror (DM), lens L1 (f = 75 mm), 1 mm deionized water jet, lens L2 (f = 50 mm), Filter 2 (band-pass filter, FBH 720-20), Spectrometer (Compact CCD spectrometer, CCS200), Lock-in amplifier system (Stanford research system, SR570 and SR830).

As shown in Figure 4, 800 nm fundamental-wave pulses were generated from a Ti:sapphire regenerator system (100 fs, 3.5 mJ, 1000 Hz). These pulses were then split into two beams using a beam splitter (BS) with an energy ratio of 3:7. Subsequently, an intense 400 nm, 100 fs pulse with energy ranging from 5 to 100 µJ per pulse was used as the pump pulse, which was generated through frequency doubling of the first 800 nm, 100 fs beam. In the following steps, we used 400 nm, 100 fs pump pulses, which were linearly propagated through a 50 cm long water cuvette consisting of a polyvinyl chloride tube with two fused silica windows on each side. Upon exiting the water cuvette, the output 400 nm pump pulses were self-stretched to a few picoseconds. Specific pulse widths were experimentally measured, as detailed in the Supplementary Materials, and the pulse width was found to broaden to 2.50 ps. In the following, we performed a transient absorption experiment for the hydrated electrons in water (L = 1 cm) using various pumping energies with the stretched 400 nm, 2.50 ps pulse and probing with a 720 nm, 100 fs pulse around the absorption peak of the hydrated electrons [24]. Notably, the 720 nm, 100 fs probe pulse was generated using an optical parametric amplifier (OPA) system. The spatiotemporal overlap of the pump and probe pulses was precisely controlled using a dichroic mirror (DM) and an optical delay line (DL). Upon exiting the water, the output probe signal was collimated with an achromat lens L2 (f = 75 mm), and a band-pass filter 2 with a cut-on wavelength of 720 ± 20 nm (FBH 720-20) was used to filter out the 400 nm pump signal. Finally, we used a lock-in amplifier system to measure the dynamic hydrated electrons with various input pump energies in water. The measured electron density can be derived from the Lambert–Beer law ($\Delta T/T=\sigma_{a}L\rho$), where $\sigma_{a}=1.4\times 10^{-18}$ cm^2^ is the absorption cross-section of the hydrated electrons [24].

### 4.2. Experimental Measured the Forward SRS in Water

As depicted in Figure 4, for the investigation of Raman properties in water, a beam dump was employed to block the 720 nm, 100 fs probe beam before reaching the 1 cm water jet. We used a lens L1 (f = 75 mm) to weakly focus the self-stretched 400 nm, 2.50 ps into a 1 cm water cuvette. Upon exiting the water, as the spectra of the conical Raman signal were spatially dispersive, the forward signals were collimated by a lens L2 (f = 50 mm), a band-pass filter 2 with a cut-on wavelength of 460 ± 20 nm (FBH 460-20) was used to filter out the 400 nm pump signal, and the spectra were measured using a spectrometer with a neutral density filter (OD: 1.0) for attenuation. All experiments were performed at ambient temperature and pressure.

## 5. Conclusions

For this study, we employed an intense self-stretched 400 nm, 2.50 ps pump pulse focused into water. The generated excess electrons were injected into the hydrogen bond network of the water cluster, leading to the formation of saturated and stable hydrated electrons from the pulse front of the excitation. We presented the experimental observation of a weak forward SRS emission at 463 nm, corresponding to 3400 cm^−1^, which is indicative of the ordinary OH stretching vibration in the liquid phase. Additionally, an intense forward SRS emission at 460 nm in water exhibited two anomalous characteristic Raman shifts at 3260 cm^−1^ and 3355 cm^−1^, which can be attributed to the contributions of electron enhancement of the Raman cross-section and the formation of hydrated electrons in water. We determined that the formation of saturated hydrated electrons plays a key role in producing this phenomenon.

## Data Availability

Data are available on request due to restrictions, e.g., privacy or ethics.

## Supplementary Materials

The following supporting information can be downloaded at: https://www.mdpi.com/article/10.3390/molecules29061245/s1, Figure S1: Schematic of the sum-frequency generation experiment setup to measure the output pulse width. Beam splitter (BS), beta barium borate (β-BBO) crystal, Filter 1 (short-pass filter, FESH0650) delay line (DL), 50 cm-long water cuvette, dichroic mirror (DM), alpha barium borate (α-BBO) crystal, Filter 2 (short-pass filter, FESH0350), Digital Optical Power Meter (PM100D), respectively; Figure S2: The measured pulse width after propagating through the 50 cm-long water sample is 2.50 ± 0.12 ps [33].
