# Supplementary material for: Hydrated Electron Dynamics and Stimulated Raman Scattering in Water Induced by Ultrashort Laser Pulses
Source: Molecules. 2024 Mar 11;29(6):1245. doi: 10.3390/molecules29061245 (PMC10975906; doi:10.3390/molecules29061245)
Supplement: Supplementary file 1 [file molecules-29-01245-s001.zip › molecules-2890999-supplementary.pdf]

molecules

Supplementary Materials

Experimental measurements were conducted on the pump pulse width.

We experimentally measured the pulse width of the output pump pulse. As illustrated in Figure S1, 800 nm fundamental-wave pulses were generated from a Ti:sapphire regenerator system (100 fs, 3.5 mJ, 1000 Hz). These pulses were then divided into two beams by a beam splitter (BS) with an energy ratio of 3:7. Subsequently, an intense 400 nm, 100 fs pulse with energy ranging from 5 to 100 μJ per pulse was used as the pump pulse, generated from the frequency doubling of the initial 800 nm, 100 fs beam.

In the subsequent steps, the 400 nm, 100 fs pump pulses linearly propagated through a 50 cm-long water cuvette, comprised of a polyvinyl chloride tube with two fused silica windows on each side. Upon exiting the water cuvette, the output 400 nm pump pulses were self-stretched to a few picoseconds. To precisely measure the pulse widths of the output 400 nm pump pulses, we performed a pump-probe measurement by directing the output 400 nm pump pulses into an α-BBO crystal to generate the sum-frequency with the second delayed 800 nm, 100 fs probe pulses from the Ti:sapphire regenerator. The spatiotemporal overlap of the pump and probe pulses was accurately controlled using a dichroic mirror (DM) and an optical delay line (DL). A short-pass filter 2 with a cut-on wavelength of 350 nm (FESH 0350) was employed to filter out the 800 nm probe and 400 nm pump signals. A power meter was then used to measure the intensity of the generated 266 nm sum-frequency signal as a function of the delay time. Finally, Gaussian fitting was applied to the experimental data to accurately determine the pulse width of the excitation beam. In subsequent experiments, the self-stretched 400 nm pulse from the output 50 cm of water was utilized as the excitation beam.

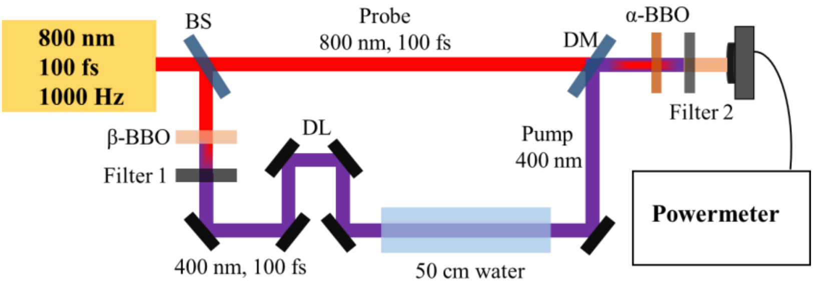

Figure S1. Schematic of the sum-frequency generation experiment setup to measure the output pulse width. Beam splitter (BS), beta barium borate (β-BBO) crystal, Filter 1 (short-pass filter, FESH0650) delay line (DL), 50 cm-long water cuvette, dichroic mirror (DM), alpha barium borate (α-BBO) crystal, Filter 2 (short-pass filter, FESH0350), Digital Optical Power Meter (PM100D), respectively.

As shown in Figure S2, the measured pulse width was broadened to 2.50 ± 0.12 ps when the input pump energy was 60 μJ. We further developed a theoretical model to confirm the experiment results. This model encompasses the Kerr self-focusing of pulsed Gaussian beams in water and employs variational methods to obtain an analytical as well as numerical solutions [1]; the calculated pulse widths with our experimental parameters agree with the measured values above.

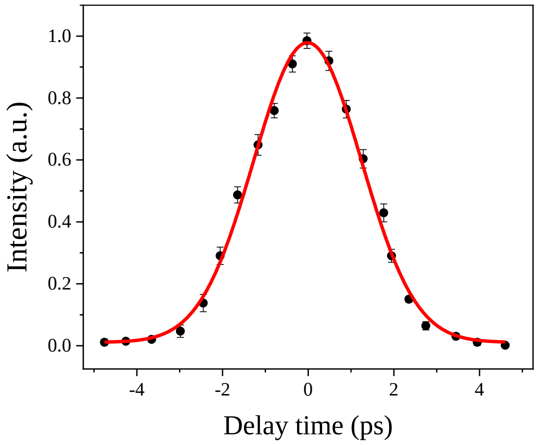

Figure S2. The measured pulse width after propagating through the 50 cm-long water sample is 2.50 ± 0.12 ps.

References

1. Chen, X.; Tang, J.; Wang, Z. Dynamic nonlinear X-waves for femtosecond filamentation in Kerr media. *Phys. Rev. A.* 2024, *in preparation*.
